# A foci cohort analysis to monitor successful and persistent foci under Thailand’s Malaria Elimination Strategy

**DOI:** 10.1186/s12936-021-03648-8

**Published:** 2021-02-27

**Authors:** Prayuth Sudathip, Suravadee Kitchakarn, Jui A. Shah, Donal Bisanzio, Felicity Young, Deyer Gopinath, Niparueradee Pinyajeerapat, David Sintasath, Cheewanan Lertpiriyasuwat

**Affiliations:** 1 Division of Vector Borne Diseases, Department of Disease Control, Ministry of Public Health, Nonthaburi, Thailand; 2 Inform Asia: USAID’s Health Research Program, RTI International, Bangkok, Thailand; 3 Division of Epidemiology and Public Health, School of Medicine, University of Nottingham, Nottingham, UK; 4 World Health Organization, Nonthaburi, Thailand; 5 U.S. President’s Malaria Initiative, United States Agency for International Development (USAID), Regional Development Mission for Asia, Bangkok, Thailand

**Keywords:** Elimination, Surveillance, 1-3-7 strategy, Foci investigation and response

## Abstract

**Background:**

Thailand’s success in reducing malaria burden is built on the efficient “1-3-7” strategy applied to the surveillance system. The strategy is based on rapid case notification within 1 day, case investigation within 3 days, and targeted foci response to reduce the spread of *Plasmodium spp.* within 7 days. Autochthonous transmission is still occurring in the country, threatening the goal of reaching malaria-free status by 2024. This study aimed to assess the effectiveness of the 1-3-7 strategy and identify factors associated with presence of active foci.

**Methods:**

Data from the national malaria information system were extracted from fiscal years 2013 to 2019; after data cleaning, the final dataset included 81,012 foci. A Cox’s proportional hazards model was built to investigate factors linked with the probability of becoming an active focus from 2015 to 2019 among foci that changed status from non-active to active focus during the study period. We performed a model selection technique based on the Akaike Information Criteria (AIC).

**Results:**

The number of yearly active foci decreased from 2227 to 2013 to 700 in 2019 (68.5 %), and the number of autochthonous cases declined from 17,553 to 3,787 (78.4 %). The best Cox’s hazard model showed that foci in which vector control interventions were required were 18 % more likely to become an active focus. Increasing compliance with the 1-3-7 strategy had a protective effect, with a 22 % risk reduction among foci with over 80 % adherence to 1-3-7 timeliness protocols. Other factors associated with likelihood to become or remain an active focus include previous classification as an active focus, presence of *Plasmodium falciparum* infections, level of forest disturbance, and location in border provinces.

**Conclusions:**

These results identified factors that favored regression of non-active foci to active foci during the study period. The model and relative risk map align with the national malaria program’s district stratification and shows strong spatial heterogeneity, with high probability to record active foci in border provinces. The results of the study may be useful for honing Thailand’s program to eliminate malaria and for other countries aiming to accelerate malaria elimination.

## Background

Thailand’s 90-year history of implementing malaria interventions [1] has resulted in significant progress in reducing the incidence of malaria to < 1 case per 1,000 people. By 2018, 81 % (55.9 million) of the population was living in malaria-free areas, 17 % (11.6 million) in low-transmission areas (0–1 cases per 1000 people), and 2 % (1.5 million) in high-transmission areas (> 1 cases per 1000 people) [2]. The malaria-free areas are concentrated in the center of the country, with most remaining malaria transmission foci along its borders with Cambodia, Myanmar, and Malaysia. Thailand’s success is built on an efficient surveillance system that provides rapid case notification, case investigation, and targeted interventions to reduce the spread of Plasmodium spp. These strategies have brought the country closer to its goal of malaria elimination.

Aligned with the World Health Organization’s (WHO’s) Strategy for Malaria Elimination in the Greater Mekong Subregion (GMS) 2015–2030 [3], Thailand’s Ministry of Public Health (MOPH) introduced the National Malaria Elimination Strategy 2017–2026 (NMES) with the target to achieve malaria-free status by 2024 [4]. As Thailand pivoted from a malaria control program to a malaria elimination program with the adoption of the NMES in 2016, the national malaria program, known as the Division of Vector Borne Diseases (DVBD) in the Department of Disease Control (DDC) of the MOPH, transitioned its malaria surveillance system to a case-based system. The DVBD also implemented malaria risk stratification at the district level and foci mapping at the sub-village level, both of which are updated each year. This stratification strategy has driven steady progress in reducing the number of reported active foci with ongoing autochthonous transmission [5].

Thailand’s surveillance system is based on the “1-3-7” strategy that was successfully implemented in China [6], which helped China reach zero indigenous cases in 2019 [2]. This strategy requires that, for each malaria case, notification occurs within 1 day of diagnosis, case investigation is completed within 3 days, and focus investigation and response are completed within 7 days to interrupt local transmission. Cases are reported in near real time through the DVBD’s Malaria Online system that serves as the routine health information system for malaria [7]. The case investigation collates the epidemiological, entomological, and socio-demographic information of each patient. After these data are analyzed, district-level Surveillance and Rapid Response Teams (SRRTs) deploy a tailored package of interventions for active case detection and vector control within 7 days.

Currently, most malaria cases are caused by *Plasmodium vivax (P. vivax)* and *Plasmodium falciparum* (*P. falciparum*) infections, with the relative proportion due to *P. falciparum* steadily decreasing as the DVBD has made progress in reducing malaria burden [5]. Whereas *P. vivax* infections can remain latent over long periods, *P. falciparum* parasites are responsible for the most acute febrile infections and—more importantly—have shown signs of artemisinin resistance [8]. The DVBD’s strategy targets both parasites, with a special urgency to eliminate *P. falciparum* parasites to address the threat of declining clearance rates among available first-line drugs [9].

The high human mobility across the GMS influences the DVBD’s tailored malaria elimination strategies to ensure that all populations in active foci at the borders receive high-quality case management [10]. For example, along Thailand’s eastern border with Cambodia, where resistance to a number of antimalarial drugs including sulfadoxine-pyrimethamine, artesunate-mefloquine, and—more recently—dihydroartemisinin-piperaquine has emerged [11, 12], the DVBD’s surveillance now includes monitoring drug efficacy to ensure parasite clearance of confirmed cases. The border between northwestern Thailand and Myanmar, at Tak province, is characterized by high human mobility due to needed employment, healthcare, or other social services. In this area, the DVBD provides malaria interventions for migrant populations to reduce the risk of outbreaks by imported cases [13]. Thailand’s southern provinces bordering Malaysia have long experienced political disruptions that complicate the effective delivery of essential health services, including malaria testing and treatment [14, 15]. To address these challenges, the DVBD partners with the Armed Forces Research Institute of Medical Sciences and the Royal Thai Army to safely and efficiently access communities at risk for malaria.

Since 2009, Thailand has conducted varied forms of case investigation, case classification, and foci investigation for malaria control and elimination [16]. With the adoption of the NMES, these interventions were refined to include a strict temporal requirement and to feed data into Malaria Online [17]. However, a robust assessment of targeting transmission foci has not yet been performed. This study aims to assess the effectiveness of the 1-3-7 strategy applied to malaria stratification by following a cohort of active and non-active foci from 2015 to 2019. The results of the study may be useful for further honing Thailand’s program to drive elimination by 2024 and prevent backsliding. The findings may also be helpful for other GMS countries aiming to accelerate malaria elimination.

## Methods

### Description of the current 1-3-7 strategy

The 1-3-7 strategy applied by Thailand’s MOPH requires that, for each identified malaria case, notification occurs within 1 day of diagnosis, case investigation is completed within 3 days, and focus investigation (if required) is completed within 7 days to interrupt local transmission. The DVBD classifies all sub-villages or villages, hereafter referred to as foci, into four levels based on source of malaria exposure (autochthonous/imported) and habitat characteristics (unsuitable/suitable for transmission).

Thailand has been using a classification system for foci for several years, throughout both the malaria control and malaria elimination phases. Prior to 2016, strata were divided into two transmission areas (A) and two non-transmission areas (B) [18]. In addition to these two areas, the DVBD also classified pre-integration and integration areas that had sustained low-risk status for a minimum of 3 years [18]. Thailand’s former and current foci classifications are summarized in Table 1.

**Table 1:** Foci classification in Thailand

Foci classification	Pre-2016 definition	Current definition
A1	Perennial transmission village or hamlet where indigenous cases are reported at least 6 months out of the year	*Active foci*: Reported indigenous transmission in the current year
A2	Periodic transmission area village or hamlet where indigenous cases are reported fewer than 6 months out of the year	*Residual non-active foci*: No indigenous cases in the current year but with indigenous cases in the previous 3 years
B1	High-risk village or hamlet without transmission for a minimum of 3 years, but adult vectors or larvae are present or conditions are favorable for breeding	*Cleared foci but receptive*: No indigenous transmission in at least 3 years, but suitable environmental for vector *Anopheles spp.* mosquitoes
B2	Low-risk village or hamlet without transmission for a minimum of 3 years, and no presence of adult vectors or larvae and unfavorable conditions for breeding	*Cleared foci but not receptive*: No indigenous transmission in at least 3 years, but unsuitable environmental for vector *Anopheles spp.* mosquitoes

Thailand’s dynamic foci classification process is a combination of real-time classification and systematic annual cross-checking and re-classification. Any indigenous case will trigger an A2, B1, or B2 focus to revert to A1 status immediately upon completion of case classification. All data are re-reviewed as part of the foci re-classification and verification process that occurs at the end of each fiscal year. During this 2-month process, interventions are planned according to each focus classification. Details of the intervention package for each stratum are summarized in Table 2.

**Table 2:** Intervention packages for each focus classification stratum

Domain	Active foci (A1)	Residual non-active foci (A2)	Receptive foci (B1)	Non-receptive foci (B2)
Surveillance	1-3-7 strategy Passive case detection (PACD), 2 rounds Foci investigation (persistent indigenous)	1-3-7 strategy PACD, 1 round Foci investigation (if active)	1-3-7 strategy	1-3-7 strategy
Diagnosis	Community (rapid diagnostic test [RDT]/microscopy) Hospitals (microscopy)	Community (RDT/microscopy) Hospitals (microscopy)	Hospitals (microscopy)	Hospitals (microscopy)
Treatment and follow up	Supervise treatment Follow up	Supervise treatment Follow up	Supervise treatment Follow up	Supervise treatment Follow up
Vector control	Insecticide-treated nets (ITNs) (≥ 90 % coverage)	ITNs (≥ 90 % coverage)		

For indigenous cases identified in a sub-village or village with the presence of relevant vector species (i.e., foci classified as A1, A2, or B1), SRRTs launch reactive case detection (RACD). SRRTs collect blood from all household members from the index case and from all neighbors within a radius of 1 kilometer, aiming for at least 50 samples or 10 households. The DVBD also coordinates appropriate health education and vector control measures, deploying insecticide-treated nets, insecticide-treated hammocks, and indoor residual spraying.

### Data dictionary

Data were extracted from Malaria Online, Thailand’s national malaria information system, on a cohort of 90,718 distinct foci with at least one data entry from fiscal years 2013 to 2019 (i.e., October 1, 2012, to September 30, 2019). The dataset contained the following variables of interest: focus classification by year (A1, A2, B1, and B2), province, sub-village or village population, presence of imported and autochthonous cases, proportion of *P. falciparum* infections among confirmed cases, bednet distribution, and indoor residual spraying (IRS) coverage.

The dataset also included information about the 1-3-7 strategy response performance per focus: the number of cases notified within 24 hours, the number of cases investigated within 3 days, the number of cases needing RACD, and the number of RACDs performed within 7 days. Using this information, we calculated the fraction of cases for which case management was not delayed. We classified 1-3-7 strategy compliance by four levels: <40 %, 40–60 %, > 60–80 %, and > 80 %.

We recoded all sub-village and village foci classification performed before 2016 using the classification adopted after 2015 to ensure consistency in definitions and to facilitate comparisons across the study period. We also checked that each focus classified after 2015 was correctly assigned to the appropriate focus group each year by cross-checking assigned foci with the presence of indigenous cases. Any incongruency found in the dataset was corrected and, as possible, missing characterizations were estimated using case reporting time series. The final dataset reflected standardized current definitions for each of the four foci classifications.

After filtering for sub-villages and villages with complete timelines from 2013 to 2019, the final dataset included 81,012 distinct foci. An alluvial graph was used to represent the dynamicity of the status changes of foci during the study period.

### Forest disturbance

Because malaria transmission in Thailand has been associated with deforestation [19], information about forest disturbance was also included in the analysis. We downloaded raster data about forest disturbance on a global scale from 2015 to 2019 [20]. The values reported in the data create a disturbance index indicating the level of disturbance on a scale from 0 (no disturbance) to 17 (highest level of disturbance) at a 25-meter resolution. We calculated the mean annual disturbance index for each province.

### Statistical modeling

A Cox’s proportional hazards model (Cox’s model) [21] was built to investigate factors linked with the probability of becoming an active (i.e., A1 status) focus from 2015 to 2019. All villages that changed status from non-active to active focus during the study period were included in the model. The model used the formula, Prob_A1_ = STATUS_y−1_ + A1_2013 − y_ + PF-RATIO + INTERVENTIONS + CASE_MANG_1 − 3−7_ + FOREST_DIST + POP + PROVINCE_random_, where Prob_A1_ is the probability of a non-active focus to become an active focus, STATUS_y−1_ is the focus status of the previous year, A1_2013 − y_ is the number of previous years during which the village was an active focus, PF-RATIO was the ratio of *P. falciparum* among all malaria cases, INTERVENTIONS was a dichotomous variable (1,0) indicating if vector control interventions (bednet distribution and IRS) were performed during the year, CASE_MANG_1 − 3−7_ is the percentage of cases managed without delays, FOREST_DIST is the mean annual disturbance index calculated for each province, and POP is the population of the village used as an adjusting factor in the model. We included province (PROVINCE_random_) as a random effect to account for province characteristics that were not captured by the other variables of the model. We performed a model selection technique based on the Akaike Information Criteria (AIC) [22].

## Results

**Fig. 1:**
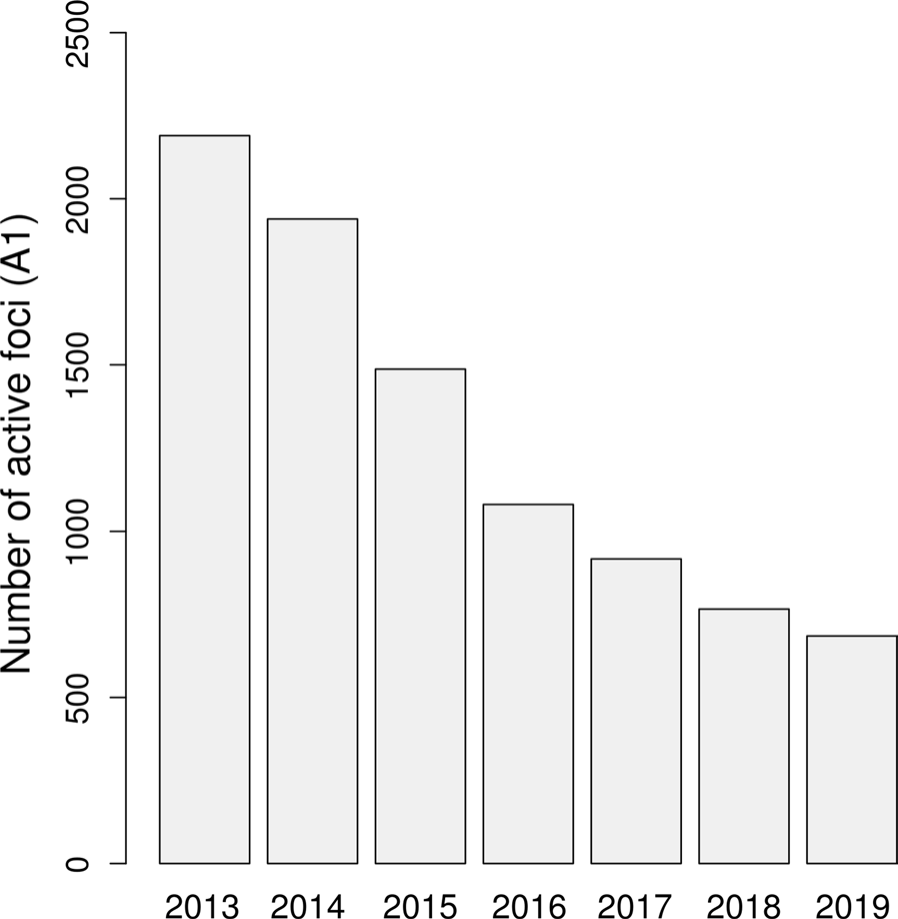
Number of reported active foci (A1) in Thailand from 2013 to 2019

Among the villages included in the analyses, a cumulative 9,230 status reports identified active foci from 2013 to 2019. The number of yearly active foci (i.e., A1) decreased by 68.5 %, from 2,227 to 2013 to 700 in 2019, with the highest reduction recorded during 2016 (Fig. 1; Table 2). Accordingly, the number of non-active foci (i.e., A2, B1, and B2) steadily increased by 1.9 %, from 78,785 to 2013 to 80,312 in 2019 (Table 3). During the study period, the number of autochthonous cases reported from the study villages declined by 78.4 %, from 17,553 to 3,787. The mean number of cases per active foci dropped from 7.3 to 2013 (median = 2, interquartile range = 1–5, maximum = 454) to 5.4 in 2019 (median = 2, interquartile range = 1–6, maximum = 103).

**Table 3:** Number of villages per focus classification from 2013 to 2019

Status	2013	2014	2015	2016	2017	2018	2019
A1	2227	1980 (− 11.1 %)	1507 (− 23.9 %)	1088 (− 27.8 %)	933 (− 14.2 %)	795 (− 14.8 %)	700 (− 11.9 %)
A2	4794	4853 (+ 1.2 %)	2907 (− 40.1 %)	3028 (+ 4.2 %)	2325 (− 23.2 %)	1681 (− 27.7 %)	1297 (− 22.8 %)
B1	14,206	14,281 (+ 0.5 %)	16,587 (+ 16.1 %)	16,682 (0.5 %)	16,592 (− 0.5 %)	16,806 (+ 1.3 %)	14,840 (− 11.7 %)
B2	59,785	59,898 (+ 0.2 %)	60,011 (+ 0.2 %)	60,214 (+ 0.3 %)	61,162 (+ 1.5 %)	61,730 (+ 0.9 %)	64,175 (+ 3.9 %)

The percentage of change since the previous year is reported in parentheses

**Fig. 2:**
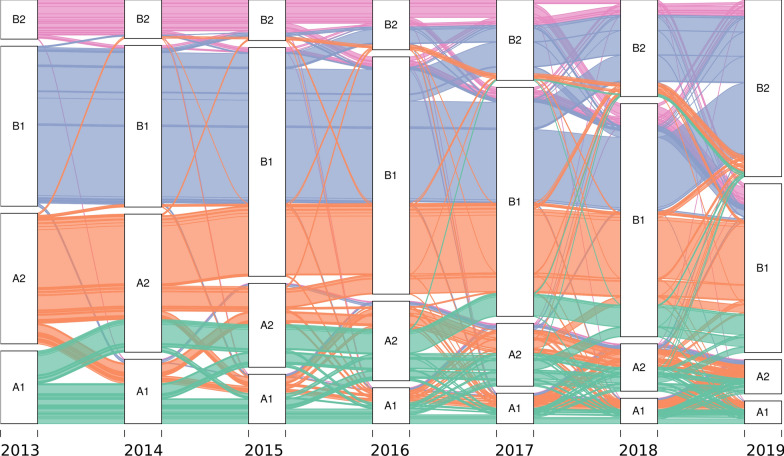
Status changes of villages from 2013 to 2019. Figure does not include B1 and B2 villages that did not change their focus status during this period

In 2019, 73,991 villages were cleared foci (91.3 %), of which 65,588 (80.9 %) were characterized as B2 and 13,801 (17.0 %) as B1. During the study period, 2125 (2.6 %) villages became active foci and 148 were persistent foci (0.2 %). Among the regression to active foci, A2 foci accounted for the major fraction equal to 1,610 villages, followed by B1 with 432 villages, and B2 with 83 villages (Fig. 2).

The Cox’s model that included all the variables was identified as the best model. The results of the model showed that foci in which interventions were required were 18 % more likely to become an A1 focus (Table 3). Increasing efficiency of case management, following the 1-3-7 strategy, has a protective effect, thereby reducing the probability of a focus becoming an active focus (Table 3). Although a level of compliance > 40 % reduces the probability of autochthonous cases, this effect was statistically significant for compliance > 80 %. This level of compliance showed a 22 % reduction of the risk.

**Fig. 3:**
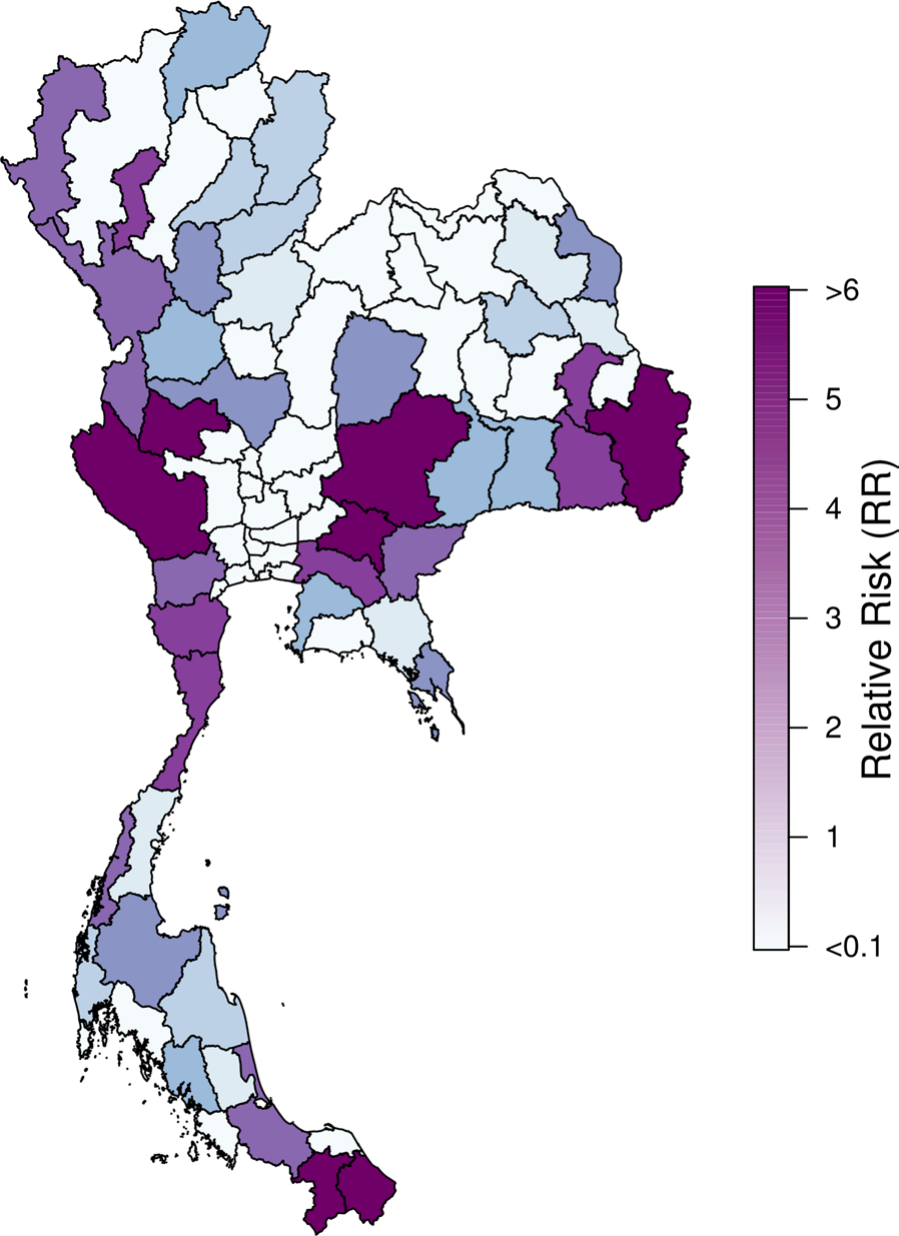
Map of relative risk of PROVINCE_random_ obtained from the Cox’s hazard model

The status of a focus in previous years has an impact on the risk of its becoming an active focus. Villages that had been an A1 focus in the past were more likely to become or remain an A1 focus (Table 4). A2, B1, and B2 foci based on the previous year’s classification had a lower probability of reporting autochthonous cases and being classified as A1 (Table 4). The presence of infection due to *P. falciparum* increased the risk of finding autochthonous cases. Foci in provinces with a high level of forest disturbance showed an increased probability of becoming active foci, but this was not statistically significant. The relative risk linked to the province showed that villages in border provinces with Lao People’s Democratic Republic, Cambodia, Myanmar, and Malaysia had a high probability to be classified as A1 (Fig. 3).

**Table 4:** Results of the Cox’s hazard model

Variable	Type of variable	Relative risk (95 % CI)
INTERVENTIONS	Dichotomous	1.18 (1.15, 1.21)*
CASE_MANG_1−3−7_ (Ref: <40 %)		
40–60 %	Ordinal	0.98 (0.92, 1.06)
>60–80 %	Ordinal	0.95 (0.87, 1.05)
>80 %	Ordinal	0.78 (0.69, 0.91)*
A1_2013 − y_	Discrete	1.23 (1.19–1.27)*
STATUS_y−1_ (Ref: A1)		
A2	String	0.24 (0.2, 0.26)*
B1	String	0.06 (0.05, 0.07)*
B2	String	0.02 (0.01, 0.05)*
FOREST_DIST	Discrete	1.18 (0.95, 1.35)
PF-RATIO (Ref: <20 %)		
20–40 %	String	1.30 (1.17–1.45)*
>40–60 %	String	1.15 (1.04–1.27)*
>60–80 %	String	1.19 (1.08–1.33)*
>80 %	String	1.17 (1.09–1.26)*

*Indicates statistical significance at the *p* < 0.05 level

The population of each focus was used as an adjusting factor in the model. Relative risk linked to PROVINCE_random_ is reported in Fig. 3.

## Discussion

The results of our study show a substantial reduction in the number of active malaria foci (A1) recorded since 2013 in Thailand. This reduction was linked with national strategies actuated to manage active foci, including the full implementation of the 1-3-7 surveillance strategy to target persistent active foci. Our analyses also identified factors that favor regression of non-active foci (A2, B1, and B2) to active foci (A1), including deforestation and lack of timely interventions. Because the AIC selected the best model, all variables included in the model contribute to the results, regardless of status at the level of statistical significance chosen for this study.

The number of active foci rapidly declined from 2013 to 2019, highlighting how the interventions implemented by the DVBD were able to reduce autochthonous transmission. The model results showed that rapid assessment of cases, based on the 1-3-7 strategy, reduced the probability of becoming active foci; furthermore, adherence to the strategy’s timelines resulted in a reduced probability of malaria transmission in the foci. The 1-3-7 strategy has been implemented in other countries approaching malaria elimination in Asia, where it is successfully reducing malaria burden [23, 24]. Despite the substantial reduction of active foci documented, 148 foci remained active for the full study period and may warrant additional studies.

Our model and relative risk map align well with the DVBD’s current district stratification and shows strong spatial heterogeneity, with high probability to record active foci in provinces bordering Myanmar, Cambodia, and Malaysia [5]. The occurrence of malaria transmission in these areas is a key challenge in the GMS’s quest for elimination [13, 26], and the persistence of active foci can be linked to factors associated with population behavior and movement. Migrant populations are hard to reach and cover with interventions due to their movement between countries and the remoteness of their settlements. As Thailand’s DVBD continues to drive down malaria burden and the foci map continues to shrink, it is likely that remaining cases will be further concentrated in the hardest to reach areas and populations. Many migrants across the GMS work in forested areas that correlate with malaria hot-spots [19, 27]. Our results show that foci in provinces with a high degree of forest disturbance had a higher probability of becoming active foci compared to foci in provinces with low forest disturbance. This further compounds the complexity of reaching target populations in border provinces; therefore, continued partnership among GMS countries will be essential for achieving and maintaining regional elimination.

Future analyses could incorporate additional geospatial components to a patient-based model to determine if B1 or B2 villages or forested areas in close proximity to A1 or A2 villages could have quantifiable risk factors. This type of model would also allow us to calculate geospatial disease patterns, connecting patients and villages with distance to forests and facilities. To prevent the risk of imported cases triggering local transmission in border areas, the DVBD could also conduct further research into examining whether a relationship exists between the number of imported cases and the risk of becoming an A1 focus. This analysis could also categorize imported cases by source (i.e., outside district, outside province, or outside country) and more closely examine the exact transmission areas.

Foci with a highly mobile population often experience a high rate of parasite introduction combined with low bednet coverage [28]. These factors can make it difficult for programs to reduce malaria transmission. Our results showed that foci in Thailand identified as needing bednet distribution and IRS were more likely to be identified as active foci. Maintaining a high level of coverage of these proven interventions is important to protect people from malaria in areas with residual transmission [25]. Thus, to reach the target of no active foci by 2021, the DVBD may consider accelerating and concentrating resources in these areas to both maintain high intervention coverage and to address bottlenecks in intervention distribution and use. Because the DVBD’s target for bednet coverage is high—for 90 % of the population in A1 foci to have access to a bednet—further analyses could also model whether a lower coverage threshold would be sufficient to see continued gains.

Across the GMS, foci and case classifications are based on the residence of a patient rather than the point of transmission. In a country such as Thailand that is approaching malaria elimination where cases are now concentrated within highly mobile communities, this method of classification may skew geospatial results. Although every focus has mapped boundaries and the majority of transmission occurs within foci boundaries, Thailand may need to explore innovative ways to address transmission that falls outside of foci boundaries. The DVBD is making efforts to better understand the source location of new infections, and these data are expected to be available soon.

The presence of *P. falciparum* infections in a previous year was associated with increased risk of autochthonous cases. Thailand has robust policies and budgets in place to address and eliminate *P. falciparum* parasites, with only 88 active foci with *P. falciparum* infections reported this year [5]. Infections caused by *P. falciparum* are more likely to be symptomatic compared to *P. vivax* infections, making them easier to identify for clinicians and public health responders [29, 30].

The DVBD has embraced WHO’s recommendation to transform surveillance into a core intervention [31]. However, although the global malaria community has explained what comprises a strong surveillance system, there are no clear definitions and tools for countries to measure and monitor the capacity of the surveillance system to accurately capture and report all facility-based and community malaria cases [32, 33]. It may be useful to conduct supplementary research or support quality assurance mechanisms to ensure that Thailand’s robust surveillance system is capturing all cases from these hard-to-reach populations as the elimination goal nears and to ensure that public health personnel, such as SRRTs, are adequately trained and resourced.

Further analyses could also include political and social factors that affect malaria transmission. For example, Yala province has long experienced political and social unrest that has complicated delivery of health services, trust in health care providers, and coverage of key malaria interventions [15]. Prachinburi and Yasothon provinces receive high numbers of migrant workers and, in Yasothon, there are military personnel who have moved frequently for assignments. These three provinces were associated with higher relative risks for harboring A1 foci. It therefore will be important for the DVBD to continue targeting key populations such as migrants and military personnel.

Movement between A1 and A2 status is frequent, which may be unsurprising due to Thailand’s sensitive foci classification criteria: having just one indigenous case reverts a focus from A2 to A1 status. To track more substantial, longer-term trends, the DVBD could consider revising its classification system to allow some margin in foci classification. Additionally, the DVBD could explore new activities to interrupt transmission, such as focal mass drug administration (paired with glucose-6-phosphate dehydrogenase [G6PD] testing), building on experience gained by GMS countries [34].

It is important for the DVBD to be prepared for the emergence of new foci due to the impact of the novel coronavirus disease (COVID-19). Under guidance of the MOPH’s DDC, Thai policymakers swiftly responded to the epidemic in early 2020 and took action, including communicating risks, restricting movement and gatherings, conducting strong surveillance and contact tracing, and closing internal borders [35, 36]. The public also took corresponding action—such as leaving Bangkok, which reported a high number of COVID-19 cases but does not have indigenous malaria transmission—for other provinces with higher malaria burden [37, 38]. This population redistribution, coupled with potential behavioral changes, may change the foci map in 2020 and beyond.

### Limitations

Although this study presents informative findings, it does have limitations. The study does not include the environmental characteristics of the villages. There is high variability in village environments, but to appropriately account for this when modeling, high-resolution data that were not included in the dataset used for this study would be needed. We also could not consider population movement across the country that could be a main driver of malaria spread because high-resolution data on migration were unavailable for the study period.

## Conclusions

The study shows that Thailand’s 1-3-7 strategy is useful to reduce autochthonous transmission, preventing non-active foci from becoming active foci. The results identified factors linked to the occurrence of active foci, which can help the DVBD to improve its surveillance and response system by targeting specific populations and areas. These results may also contribute to a future predictive model that highlights foci at risk of reverting to A1 status. The effectiveness of the 1-3-7 strategy adopted in Thailand could serve as an example for other countries in the GMS aiming to accelerate malaria elimination.

## Data Availability

The dataset supporting the conclusions of this article is available in the Malaria Online repository, http://malaria.ddc.moph.go.th/.

## Abbreviations

A1: Active foci; reported indigenous transmission in the current year
A2: Residual non-active foci; foci with no indigenous transmission in the previous 3 years
AIC: Akaike Information Criteria
B1: Cleared foci but receptive; no indigenous transmission in at least 3 years, but: suitable environmental for vector *Anopheles spp.* mosquitoes
B2: Cleared foci but not receptive; no indigenous transmission in at least 3 years, but: unsuitable environmental for vector *Anopheles spp.* mosquitoes
COVID-19: Coronavirus disease 2019
DDC: Department of Disease Control
DVBD: Division of Vector Borne Diseases
GMS: Greater Mekong Subregion
IRS: Indoor residual spraying
ITN: Insecticide-treated net
MOPH: Ministry of Public Health
NMES: National Malaria Elimination Strategy
PACD: Passive case detection
RACD: Reactive case detection
RDT: Rapid diagnostic test
SRRT: Surveillance and Rapid Response Team
USAID: United States Agency for International Development
WHO: World Health Organization
